# Cellular Localization and Regulation of Expression of the *PLET1* Gene in Porcine Placenta

**DOI:** 10.3390/ijms17122048

**Published:** 2016-12-07

**Authors:** Liu Teng, Linjun Hong, Ruize Liu, Ran Chen, Xinyun Li, Mei Yu

**Affiliations:** Key Lab of Agricultural Animal Genetics, Breeding and Reproduction of Ministry of Education and the Cooperative Innovation Center for Sustainable Pig Production, Huazhong Agricultural University, Wuhan 430070, Hubei, China; tengliu2007@126.com (L.T.); Linjun.Hong@ars.usda.gov (L.H.); lrz_2003@163.com (R.L.); 18627997896@163.com (R.C.); xyli@mail.hzau.edu.cn (X.L.)

**Keywords:** PLET1, pig placenta, trophoblast cell, alternative polyadenylation, miR-365-3p

## Abstract

The placenta expressed transcript 1 (*PLET1*) gene, which is expressed in placentas of pigs and mice, has been found to have a potential role in trophoblast cell fate decision in mice. Results of this study showed that the porcine PLET1 mRNA and protein were expressed exclusively in trophoblast cells on Days 15, 26, 50, and 95 of gestation (gestation length in the pig is 114 days), indicating that the PLET1 could be a useful marker for porcine trophoblast cells. Additionally, PLET1 protein was found to be redistributed from cytoplasm to the apical side of trophoblast cells as gestation progresses, which suggests a role of PLET1 in the establishment of a stable trophoblast and endometrial epithelial layers. In addition, two transcripts that differ in the 3′ UTR length but encode identical protein were identified to be generated by the alternative cleavage and polyadenylation (APA), and the expression of *PLET1-L* transcript was significantly upregulated in porcine placentas as gestation progresses. Furthermore, we demonstrated the interaction between the miR-365-3p and *PLET1* gene using luciferase assay system. Our findings imply an important role of PLET1 in the placental development in pigs.

## 1. Introduction

The placenta is a transiently developed organ that facilitates maternal-fetal exchange of nutrient and gas, and also serves as a barrier to protect the fetus from the maternal immune system. Thus, it is essential to maintain normal placental structure and function during pregnancy for fetal development and growth. Placenta-extensive or specific expressed genes have been proven to play important roles in regulating placenta development, and a number of those genes have been identified in humans and mice [1]. For example, Peg10 (paternally expressed 10) is an evolutionarily conserved retrotransposon-derived imprinted gene. Mice with Peg10 knockout showed an early embryonic lethal phenotype caused by the incomplete placenta formation [2]. Plac1 (Placenta-specific protein 1) is an X-linked gene, and its expression is primarily in the differentiated trophoblast cells. It was demonstrated that Plac1 is essential for normal placental development by regulating the trophoblast invasion and migration [3]. The transcription factor Gcm1 (glial cells missing-1), whose expression is mainly in trophoblast cells, is a key regulator of chorioallantoic branching morphogenesis during placental development [4]. However, in addition to the discovery of a trophoblast-specific SN1/38 antigen in pigs [5,6], the number of identified genes that are specifically or highly expressed in pig placenta is rare.

Litter size is one of the economically most important traits in pig production. Abnormalities in the placental development will lead to a decrease of litter size and an increase in preweaning mortality rates in pigs. The pig is known to have a non-invasive and diffuse epitheliochorial placenta, in which the uterine epithelial cell membrane is intact throughout gestation [7]. During Days 4–15 of gestation (normal gestation is 114 days for pigs), the conceptus (including embryo, trophectoderm, and associated extra-embryonic membranes) undergoes dramatic morphological change from spherical to filamentous forms [8]. The pig placentation is initiated when the trophoblast begins to attach to the uterine luminal epithelium around Days 15–20 of gestation. Around Days 26–30 of gestation, the non-invasive epitheliochorial placenta is established and the adhered trophoblast-endometrial epithelial bilayer starts to develop the folded structure to maximize the maternal-fetal exchange area. The primary folds are in a steady state around Day 50 of gestation. Thereafter, the folding and branching of trophoblast-endometrial epithelial bilayer becomes more complex [7,9,10,11]. To better understand the regulation mechanisms of the placentation in pig, the expression profiles of mRNA and miRNA in porcine conceptuses or placentas at different stages were investigated, and the genes, miRNAs, and miRNA/mRNA networks involved in conceptus transformation, implantation, trophoblast-endometrial epithelial bilayer development, and placenta function were reported [12,13,14,15]. On the other hand, some genes that are important for trophoblast cell function were identified. By in vitro assays, some growth factors, such as the vascular endothelial growth factor, epidermal growth factor, and insulin-like growth factor were found to be able to stimulate the proliferation and migration of the pig trophectoderm cells [16,17,18]. The transforming growth factor β could regulate the adhesive properties of the pig trophectoderm cells by inducing the expression of fibronectin [19,20]. Furthermore, several genes that are expressed by the pig conceptus and placenta have been found to be involved in regulation of the placenta development. For example, the osteopontin plays critical roles in mediating the adhesion between the conceptus trophectoderm and the uterine luminal epithelium [21,22,23]. The contribution of the cathepsins (CTSB and CTSL1) to the tissue remodeling for the apposition between maternal and fetal vasculatures has also been implicated [24]. Heparanase (HPSE) has been reported to be expressed in porcine placenta, recently, we also found that HPSE may be involved in the placental folds development [25,26]. However, the molecular mechanisms underlying the porcine placenta development remains to be explored [27].

Porcine *PLET1* (placenta-expressed transcript 1) gene was first isolated from the porcine term placenta and conceptus cDNA libraries as well. By using RT-PCR and RNA blotting methods, Zhao et al. [28,29] confirmed that porcine *PLET1* had a higher expression level in the elongated conceptus and placenta. The cDNA sequence of porcine *PLET1* was determined to encode a protein consisting of 210-amino acids of the unknown function. Although the PLET1 was undetectable in human term placenta libraries, it also found at a high frequency in bovine and mouse placenta cDNA libraries, respectively. The in situ hybridization analysis showed that the mouse Plet1mRNA was expressed in trophoblast cells of two inner placental layers, the labyrinth and spongiotrophoblast layers [28]. A recent report revealed that mouse *Plet1* was repressed in embryonic stem cells caused by DNA methylation but expressed in trophoblast stem cells and trophoblast giant cells. Overexpression of *Plet1* could promote the differentiation towards trophoblast giant cell (and/or spongiotrophoblast), but silencing of Plet1was found to induce syncytiontrophoblast formation [30]. These findings suggest an important role of PLET1 in trophoblast cell lineage determination and placentation. Due to the findings that the regulatory mechanism responsible for placental expression of related genes may not be conserved [1], the function of PLET1 on porcine placenta development remains to be understood.

Therefore, our study demonstrated the cellular location pattern of the PLET1 in porcine conceptus and placenta during periods of placenta development (Days 15, 26, 50, and 95 of gestation). In addition, a role of the alternative cleavage and polyadenylation (APA) in regulation of the PLET1 expression was investigated.

## 2. Results

### 2.1. PLET1 Gene Was Uniquely Expressed by Trophoblast Cells throughout Pig Placenta Development

**Figure 1:**
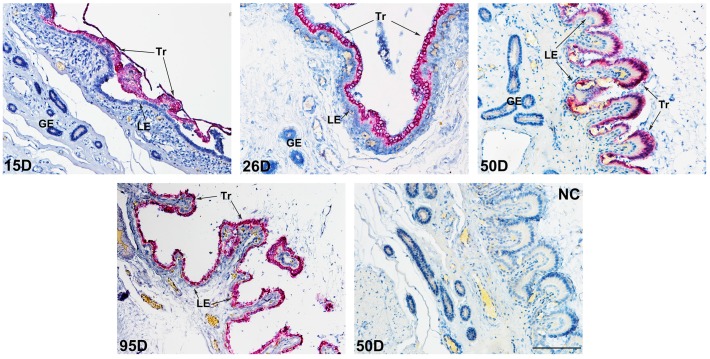
In situ hybridization analysis of PLET1 mRNA at the maternal-fetal interface in pigs on Days 15, 26, 50, and 95 of gestation. The PLET1 mRNA (rose-bengal) was uniquely localized to the trophoblast cells (Tr) at the maternal-fetal interface along the placental folds, and was lacking in the endometrial luminal epithelium (LE), glandular epithelium (GE), and stroma. The section stained with hybridization buffer A without probe was used as the negative control (NC). D: day of gestation. Scale bar = 100 μm.

**Figure 2:**
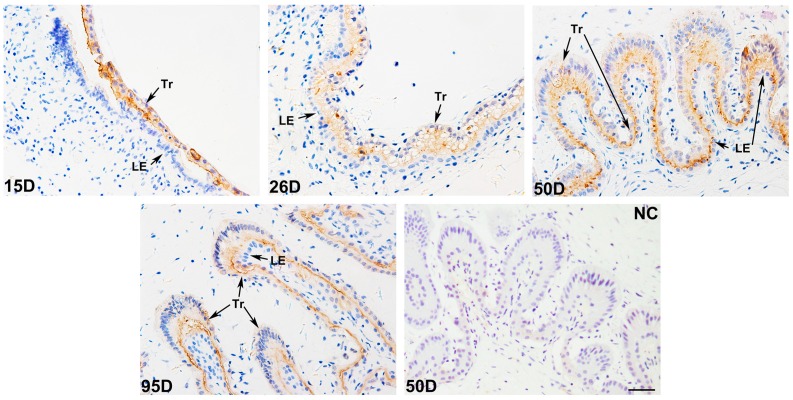
Immunohistochemical analysis of PLET1 at the maternal-fetal interface in pigs on Days 15, 26, 50, and 95 of gestation. The positive signals of PLET1 were only detected in Tr. The section stained with isotype matched normal rabbit IgG was used as the negative control. Legend: D: day of gestation. Tr: trophoblast cells; LE: luminal epithelium; NC: negative control. Scale bar = 40 μm.

Previous study showed that pig *PLET1* is highly expressed in pig placenta [28]. In this study, the in situ hybridization analysis was performed to determine the location of PLET1 mRNA at the maternal-fetal interface during pregnancy in pigs. The results revealed that the expression of *PLET1* gene was restricted to the trophoblast cells throughout the four developmental stages investigated (gestational Days 15, 26, 50, and 95). However, neither the uterine luminal and glandular epithelium nor the surrounding stroma cells were positive for PLET1 (Figure 1). Furthermore, the immunohistochemistry method was used to detect the expression pattern of PLET1 protein. Consistent with the cell-specific expression pattern of PLET1 mRNA in porcine placenta, immunoreactive-PLET1 was only detected in trophoblast cells in porcine placenta, and showed less changes in expression levels throughout the gestational periods investigated (Figure 2).

### 2.2. The Location Pattern of PLET1 in Pig Trophoblast Cells Changes as the Pregnancy Proceeds

**Figure 3:**
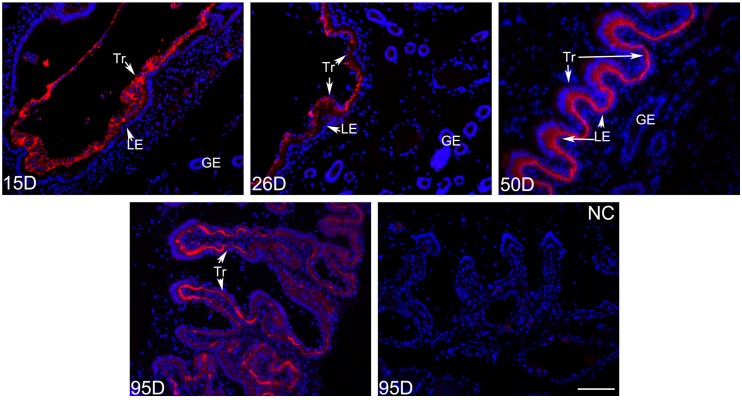
Immunofluorescence analysis of PLET1 at the maternal-fetal interface in pigs. PLET1 was identified by a red fluorescence. The section stained with normal rabbit IgG was used as the negative control. On Days 15 and 26 of gestation, the immunoreactive-PLET1 was observed in the trophoblast cells and the positive signals were located mainly in cytoplasmic region. On Days 50 and 95 of gestation, PLET1 protein gradually accumulated to the apical side of the trophoblast cells towards the uterine epithelium. Tr: trophoblast cells; LE: luminal epithelium; GE: glandular epithelium; D: day of gestation; NC: negative control. Scale bar = 100 μm.

The immunofluorescence method was used to detect the localization of PLET1 in pig trophoblast cells. On Days 15 and 26 of gestation, the PLET1 protein was present mostly in the cytoplasm of the trophoblast cells. However, on Days 50 and 95 of gestation, a change in the location of the PLET1 protein in the trophoblast cells was observed in that the PLET1protein was primarily localized at the apical side, which towards to the apical surface of the uterine epithelium. Very weak positive signals were observed in the cytoplasm of the trophoblast cells (Figure 3).

### 2.3. Two Different PLET1 Transcripts Were Identified to Be Expressed in Porcine Placenta by 3′RACE and Showed Different Expression Levels as the Placenta Developed

**Figure 4:**
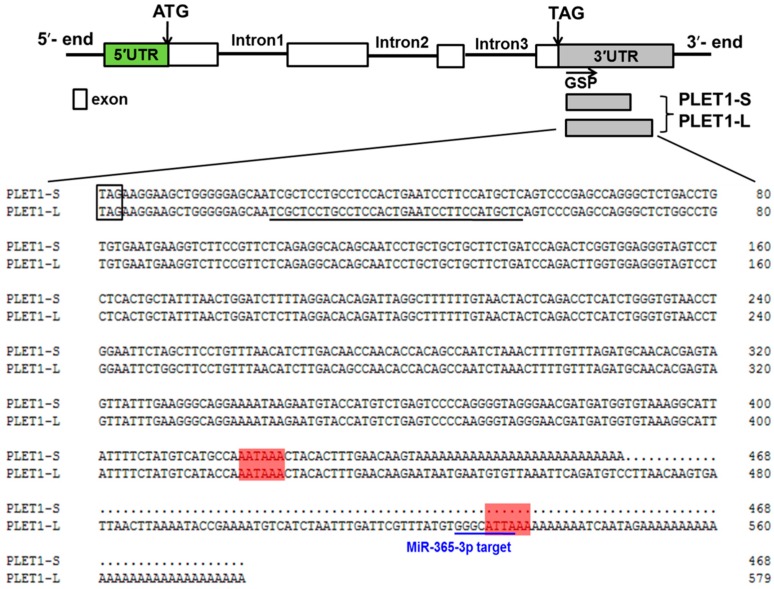
Sequence alignment of the *PLET1-S* and *PLET1-L* transcripts. The exons are shown in the box. Primer used for 3′ RCAE is underlined (black line). The stop codon is boxed. The two polyadenylation signal sequence (AATAAA and ATTAAA) are highlighted in red. The predicted miR-365-3p binding site is underlined (blue line).

The 5′ and 3′ RACE were performed to identify the positions of the 5′ and 3′ ends of the porcine *PLET1* transcript. The RNA samples used were isolated from the conceptus on gestational Day 15 and placenta (chorioallantoic tissue) on gestational Day 95. The 5′ RACE result showed one single transcript start site within *PLET1* (data not shown), which corresponded to the previously published 5′ end of the porcine *PLET1* resulted from the term placenta RNA [28]. However, by 3′ RACE, two transcripts that differ in the length of the 3′ UTRs but encode the identical protein were detected to be expressed in each of the two RNA samples. The previous in silico analysis of porcine PLET1 EST sequences has predicted the existence of two putative polyadenylation signals that are 106 bp apart [28]. Therefore, subsequent sequencing of the 3′ RACE products was performed and confirmed the existence of the two polyadenylation sites (AAUAAA and AUUAAA) within the 3′ UTR (Figure 4). We named the two transcripts as *PLET1-S* (containing the shorter 3′ UTR) and *PLET1-L* (containing the longer 3′ UTR) respectively.

**Figure 5:**
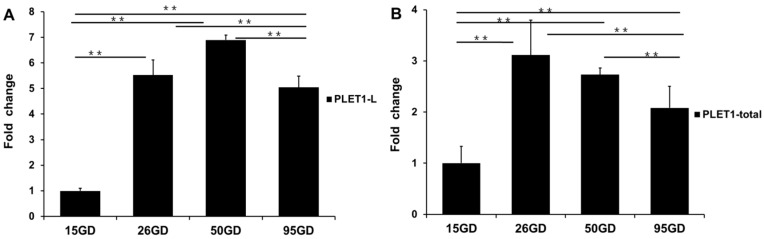
Detection of the *PLET1-L* and *PLET1-total* mRNA in conceptuses (Day 15 of gestation) and placentas (Days 26, 50, and 95 of gestation) by qRT-PCR. (**A**) The mRNA profile of *PLET1-L*; (**B**) The mRNA profile of *PLET1-total*. ** *p* < 0.01. GD: gestational day.

In order to detect the expression levels of the two transcripts during the periods of Days 15, 26, 50, and 95 of gestation in pigs, two sets of primers were designed: (1) to amplify the 3′ UTR region that is specific for the *PLET1-L* transcript (designed as PLET1-L), and (2) to amplify the overlap region of the two transcripts (designed as PLET1-total). As compared to Day 15 of gestation, the expression levels of the *PLET1-L* transcript and *PLET1-total* were significantly upregulated in placentas from Days 26, 50, and 95 of gestation compared to those in conceptuses from Day 15 of gestation (Figure 5). 

### 2.4. The Long and Short PLET1 3′ UTR Differentially Mediate the Luciferase Activity In Vitro

**Figure 6:**
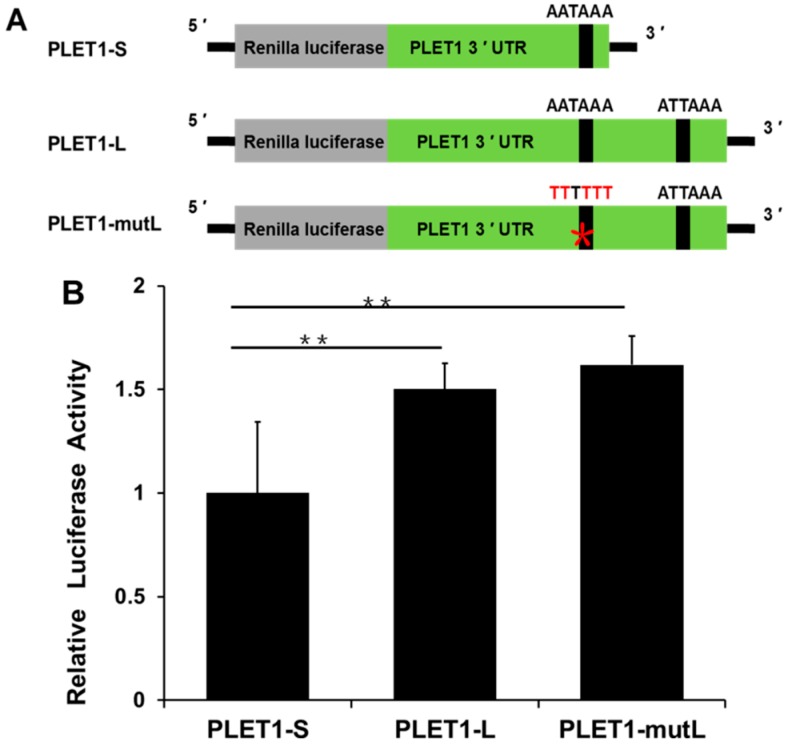
Investigation of the influence of the two *PLET1* 3′ UTRs on luciferase activity by luciferase reporter assay. (**A**) Three constructs of *PLET1* 3′ UTR luciferase reporter plasmids: PLET1-S, PLET1-L, and PLET1-mutL luciferase reporter plasmids; (**B**) *PLET1* 3′ UTR with distinct polyadenylation signals leads to the different luciferase activity. PLET1-S: *PLET1-S* 3′ UTR reporter plasmid (contains the first polyadenylation signals), PLET1-L: *PLET1-L* 3′ UTR reporter plasmid (contains the two polyadenylation signals). PLET1-mutL: 3′ UTR reporter plasmid in which the first polyadenylation signal was mutated. The *y*-axis shows the dual-luciferase activity ratio (Renilla/Firefly luciferase). The significance of differences was calculated using two-tailed *t*-test. Data are shown as mean ± SEM of three independent experiments ** *p* < 0.01.

The luciferase reporter assay was used to investigate the influence of the two *PLET1* 3′ UTRs on luciferase activity. Three luciferase reporters were generated, respectively: (1) the *PLET1-S* 3′ UTR containing only the first polyadenylation signals; (2) the *PLET1-L* 3′ UTR containing both the two polyadenylation signals; and (3) the *PLET1-mutL* 3′ UTR where the first polyadenylation signal was mutated but the second one remained. Compared to the *PLET1-L* 3′ UTR reporter, the reporter with the *PLET1-mutL* 3′ UTR had higher luciferase activity although it did not reach the level of significance. However, the *PLET1-S* 3′ UTR reporter resulted in significantly lower luciferase activity than either the *PLET1-L* 3′ UTR reporter or the *PLET1-mutL* 3′ UTR reporter (*p* < 0.01) (Figure 6).

### 2.5. Investigation of the Interaction between Pig PLET1 and miR-365-3p In Vitro

**Figure 7:**
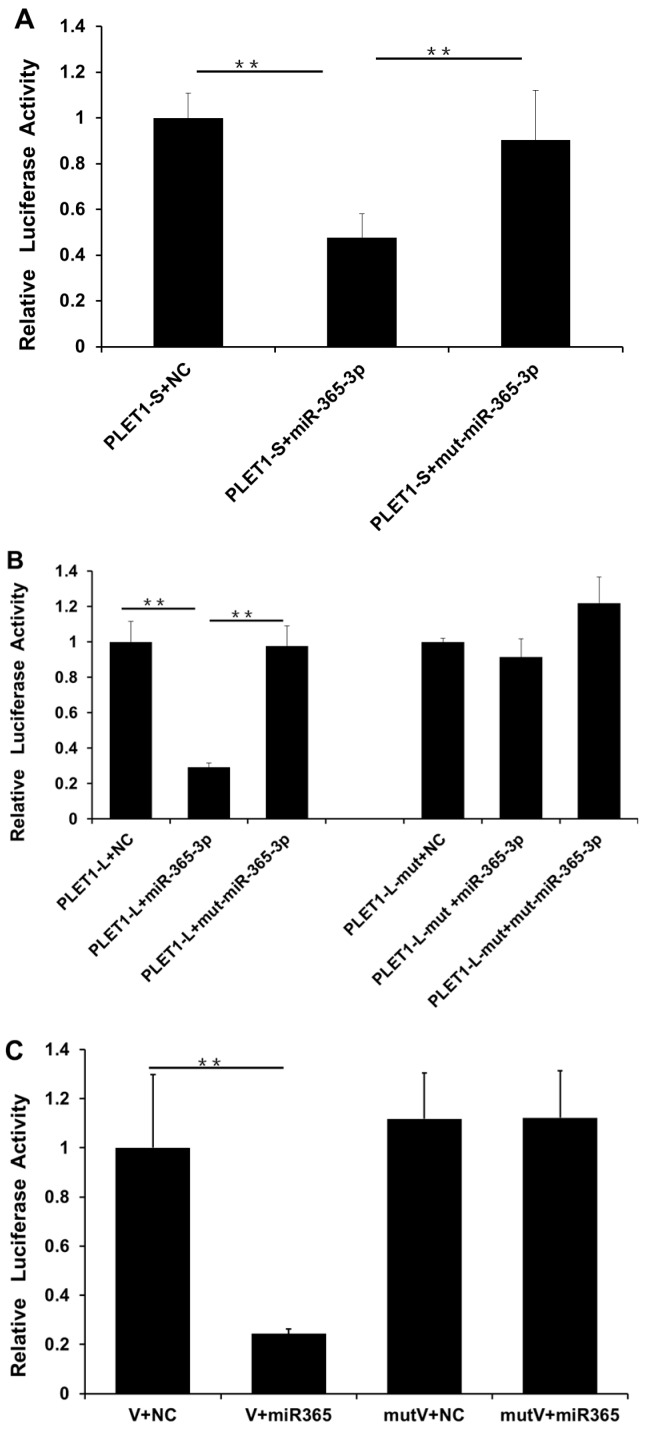
Validation of the interaction between miR-365-3p and *PLET1* 3′ UTR by luciferase assay. (**A**) Luciferase assay of cells transfected with PLET1-S luciferase vectors. PLET1-S: luciferase vector of the full length of *PLET1-S* 3′ UTR; mut-miR-365-3p: miR-365-3p mutant; (**B**) Luciferase assay of cells transfected with PLET1-L and PLET1-L-mut luciferase vectors. PLET1-L: luciferase vector of the full length of *PLET1-L* 3′ UTR; PLET1-L-mut: luciferase vector of *PLET1-L* 3′ UTR carrying mutant miR-365-3p binding site; (**C**) Luciferase assay of cells transfected with the truncated *PLET1-L* 3′ UTR luciferase vectors that contains the predicted binding site or mutated binding site. V: the truncated *PLET1-L* 3′ UTR (26 bp) luciferase vector containing the miR-365-3p seed binding site; mutV: mutant vector of the truncated *PLET1-L* 3′ UTR fragment (26 bp) containing the mutated seed binding site. Data are shown as mean ± SEM of three independent experiments. The significance of differences was calculated using two-tailed *t*-test. ** *p* < 0.01.

In order to investigate the factors mediating the expression of the two *PLET1* transcripts, the online miRNA target prediction tools (available online: http://www.microrna.org/microrna/home.do) and (available online: http://bibiserv.techfak.uni-bielefeld.de/rnahybrid/) were used to predict the binding sites of miRNAs within the two 3′ UTRs. First, four putative miRNA binding sites were found in the *PLET1* 3′ UTR; Second, we investigated the interactions between the miRNAs and their putative binding sites in vitro by using the dual-luciferase reporter system, respectively. The full length 3′ UTR of the *PLET1-L* was directly cloned into the dual luciferase reporter plasmid. Of the four miRNAs, only the miR-365-3p mimic could result in decrease in luciferase activity. It is worth noting that the 5′ end of the seed sequence of miR-365-3p matches the first 4 bp of the distal (second) core polyadenylation signal (AUUAAA) (Figure 4); Third, we validated the interaction between miR-365-3p and porcine *PLET1* by transfection of three constructs (the *PLET1-L* 3′ UTR, *PLET1-S* 3′ UTR and mutant *PLET1-L* 3′ UTR where the putative miR-365-3p binding site was mutated) into PK15 cells, respectively. We found the following: (1) The *PLET1-S* 3′ UTR luciferase reporter activity decreased ~2.0-fold in cells transfected with miR-365-3p compared to NC-miRNA, and overexpression of miR-365-3p mutant did not result in the decrease of *PLET1-S* 3′ UTR luciferase activity (Figure 7A); (2) The *PLET1-L* 3′ UTR luciferase reporter activity decreased ~3.0-fold in cells transfected with miR-365-3p compared to NC-miRNA. However, *PLET1-L* 3′ UTR luciferase activity had a similar level in cells transfected with miR-365-3p mutant compared to NC-miRNA (Figure 7B); (3) The *PLET1-L-mut* was co-transfected with NC-miRNA, miR-365-3p, and miR-365-3p mutants, respectively. As showed in Figure 7B, neither miR-365-3p nor miR-365-3p mutants could result in significant decrease in the *PLET1-L-mut* luciferase reporter activity (Figure 7B). 

Finally, we constructed the truncated wild-type plasmid containing the predicted binding site of miR-365-3p and the corresponding mutant plasmid containing the mutated seed binding site. We found that overexpression of the miR-365-3p significantly decreased the luciferase activity of the wild-type plasmid compared to that of the NC control. However, the mutant plasmid had the similar luciferase activity as the NC control (Figure 7C). In summary, these results demonstrated the interaction between the PLET1 and miR-365-3p in vitro.

### 2.6. miR-365-3p Was Differentially Expressed during Days 15, 26, 50, and 95 of Gestation in Pigs

**Figure 8:**
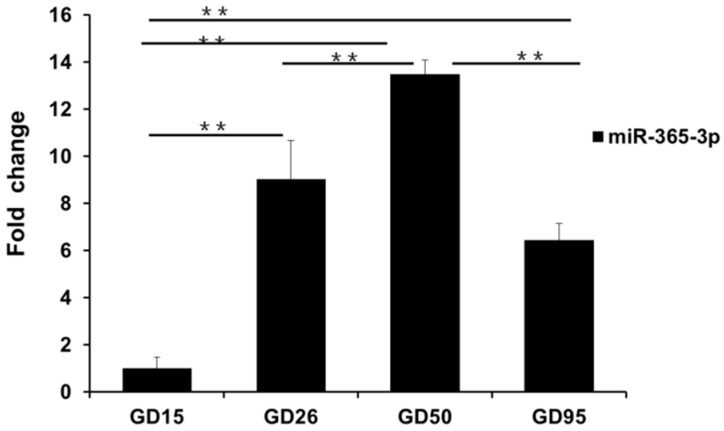
Investigation of the expression pattern of miR-365-3p in pig conceptus (Day 15 of gestation) and placenta by using qRT-PCR. Data are shown as mean ± SEM of three independent experiments. ** *p* < 0.01. GD: gestational day.

The qRT-PCR was used to detect the expression pattern of miR-365-3p in conceptus and placenta during the periods Days 15, 26, 50, and 95 of gestation. As shown in Figure 8, the miR-365-3p expression levels were significantly increased in placentas from Days 26, 50, and 95 of gestation as compared to that in conceptuses from Day 15 of gestation.

### 2.7. PLET1 Is an N-Glycosylation Modified Protein

**Figure 9:**
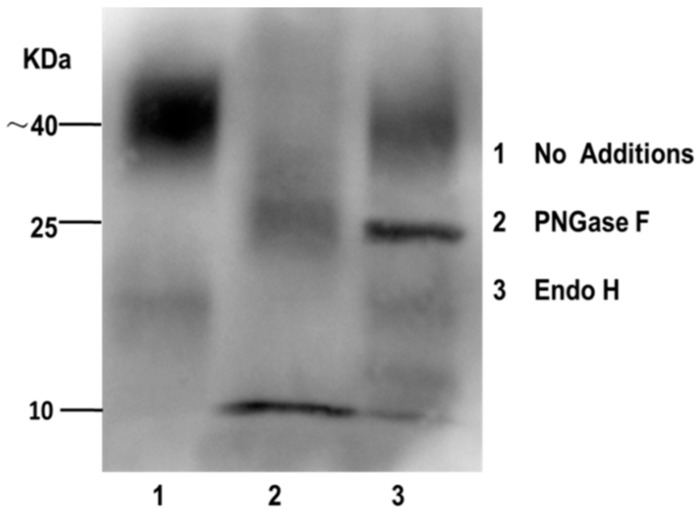
Determination of the *N*-glycosylation status of PLET1 protein by endoglycosidase treatments and western blotting. Tissue lysates were incubated with PNGaseF or EndoH independently, the extracts were then resolved by SDS-PAGE. Western blotting was probed with anti-porcine PLET1 antibody. Lane 1: Tissue lysates were in absence of PNGaseF or EndoH; Lane 2: Tissue lysates were incubated with PNGase F that removes all N-linked sugars; Lane 3: Tissue lysates were incubated with Endo H, which removes only immature, high mannose-containing, or hybrid sugars.

Sequence analysis of the *PLET1* full-length cDNA revealed the existence of an ORF of 630 bp encoding a putative protein consists of 210 amino acids with a theoretical molecular mass of 23.2 kDa. However we found that the molecular mass of PLET1 protein was about 40 kDa by western blots (Figure 9). Bioinformatics analysis predicted that the porcine PLET1 contains two *N*-glycosylation modified sites, at Asn-67 and Asn-94, respectively. So we determined the *N*-glycosylation status of PLET1 protein by incubating the placenta tissue lysates with two different glycosidases: (1) Endo H, which removes the immature high mannose-containing or hybrid sugars; and (2) PNGase F which removes all the N-linked sugars, leading to full N-linked deglycosylation of glycoproteins. A decrease in molecular mass of the PLET1 protein indicated the removal of sugars from the peptide. As showed in Figure 9, the apparent molecular mass of PLET1 protein is approximately 40 kDa, which is heavier than that of the predicted 23.2 kDa. Treatment of PLET1with the PNGase F resulted in two bands of 25 and 10 kDa respectively, and the EndoH treatment yielded mainly 25 kDa protein. Therefore the porcine PLET1 was sensitive to both Endo H and PNGase F treatment. The result indicates that the porcine PLET1 expressed in placenta was modified by *N*-glycosylation.

## 3. Discussion

Previous studies using the microarray and RT-PCR methods showed that *PLET1* gene is highly expressed in porcine placenta which consists of trophoblast cells and the stromal layer underneath the trophoblast cells [28,29,31]. In this study, we demonstrated that the expression of PLET1 mRNA and protein is restricted to the trophoblast cells in porcine placenta before (Day 15 of gestation) and after (Days 26, 50, and 95 of gestation) the establishment and development of the epitheliochorial placenta. Our data therefore extended the previous findings. In addition, the cell type-specific expression of PLET1 in placenta makes this gene a unique marker for the trophoblast cells in porcine placenta.

In pigs, the trophoblast cells start to attach to the uterine luminal epithelium around Days 15–20 of gestation. The adhered trophoblast-endometrial epithelial bilayer is formed around Days 26–30 of gestation. Thereafter the functional trophoblast-endometrial epithelial bilayer develops the very characteristic folds [7,10]. Thus, the structure and function of the trophoblast cells during the early placentation stage may be to some extent different from those after the complete structure of placenta has formed. Before the trophoblast cells and uterine epithelium are fully zipped together in pigs, the trophoblast cells exhibit increased mitotic rate together with rapid cytoskeletal reorganization and develop adhesion competency to mediate the relatively low-affinity contacts with the uterine epithelium. After the true epitheliochorial placenta has been established, the adhesive interactions become more stable [8]. Interestingly, our results revealed that porcine PLET1 was primarily present throughout the cytoplasmic region of the trophoblast cells on gestational Days 15 and 26, and then as the gestation advanced the protein was gradually accumulated to the apical position of the trophoblast cells which is adjacent to the apical surface of the uterine epithelium (Figure 3). This finding suggests a potential relationship between the PLET1 localization pattern in trophoblast cells and the changes in structure and function of the trophoblast cells during the different placentation stages. We demonstrated that porcine PLET1 is an *N*-glycosylated glycoprotein. It has been known that protein glycosylation modification is often essential for the correct cellular localization or the function of protein [32,33]. However, we found that the molecular weight of porcine PLET1 remains unchanged during the four gestational days (data not shown), implying that the glycosylation status of porcine PLET1 did not change as the gestation advanced. The finding suggests that the localization of PLET1 in porcine trophoblast cells is not dependent on its *N*-glycosylation status. However, whether the *N*-glycosylation modification of PLET1 affects its protein function in trophoblast cells is worth investigating. It has been reported that mouse Plet1 is also an orphan protein modified by *N*-glycosylation [34]. The expression of mouse Plet1 was restricted to the trophoblast cells of the labyrinth and spongiotrophoblast layers that mediate the fetal–maternal exchange and cell migration [28]. A recent report indicates that mouse Plet1 is an important regulator in directing trophoblast cell differentiation. Trophoblast cells with high Plet1 levels preferentially differentiate into cells including spongiotrophoblast [30]. The porcine placenta trophoblast layer is functionally analogous to the trophoblast cells of the labyrinth and spongiotrophoblast layers, thus suggesting a possible conserved role of PLET1 in mediating the pig trophoblast cell differentiation. On the other hand, previous findings from the stable shRNA-mediated *Plet1* gene silencing in the mouse outer root sheath keratinocytes suggest that Plet1functions in regulating hair follicle cellular migration and adhesion to collagens I and IV. In addition, the restricted distribution pattern of Plet1in the hair follicle implies that Plet1may facilitate to anchor the hair shaft to the follicle [34]. These results provided evidence to indicate that PLET1 may also have a role in mediating cellular adhesion. During the pig placental development, the accumulation of PLET1 in the apical position of the trophoblast cells may help to maintain stable adhesive interactions between the trophoblast and endometrial epithelial layers. Therefore, the role of PLET1 in the formation and stabilization of the porcine trophoblast-endometrial epithelial bilayer is worth further investigation. 

Two transcript variants of mouse *Plet1* with alternate exons 4 were identified. “Mouse 1” is a membrane-bound protein and “mouse 2” was suggested to be a secreted isoform [35]. In this study, two transcripts were observed to be expressed in the porcine placenta as determined by 3′ RACE. Sequence analysis revealed that the two transcripts (*PLET1-S* and *PLET1-L*) produce the same protein but differ in the length of the 3′ UTRs due to the alternative cleavage and polyadenylation (APA). APA is a widespread mechanism in eukaryotes that can influence the mRNA localization and stability, as well as the tissue specific expression of the mRNA transcripts [36,37,38,39]. Due to the fact that the distinct 3′ UTR isoforms presumably interact with different trans-acting factors, such as the microRNA (miRNA) and RNA-binding proteins (RBPs), the APA can also regulate the protein abundance through changes in the 3′ UTR isoform ratios [36,37,40]. Most recently, Berkovits et al. showed that CD47 produced by the long 3′ UTR isoform primarily localizes to the plasma membrane, whereas CD47 encoded by the short 3′ UTR isoform localizes to the endoplasmic reticulum. Further investigation revealed that the long *CD47* 3′ UTR can bind to RBP HuR, which recruits SET to active RAC1. These interactions enable the CD47 to localize to the plasma membrane. The finding suggests a new function of 3′ UTR in formation of protein complex by acting as scaffolds to recruit effector proteins to the site of translation and then determining the protein localization and function [41]. In this study, we observed that the location of porcine PLET1 protein within the trophoblast cells was gradually changed from the cytoplasmic region to the apical position as gestation proceeds (Figure 3). Correspondingly, the expression of *PLET1-L* transcript was detected to be upregulated as gestation progresses. Therefore, the implications of the expression of the two *PLET1* transcripts in the establishment of porcine placenta require further investigation.

Our study predicted a putative binding site of miR-365-3p in pig *PLET1-L* 3′ UTR.The in vitro luciferase assays revealed ~3.1-fold decrease of the *PLET1-L* 3′ UTR luciferase reporter activity in cells transfected with miR-365-3p mimics compared to that in cells transfected with NC-miRNA. We then developed a second *PLET1-L* 3′ UTR luciferase reporter carrying a mutant miR-365-3p binding site. The mutant cassette was the result of mutating of GGGC***ATTA*** to TAGA***ACTA*** (the mutated nucleotides are underlined, and the first four nucleotides of the second polyadenylation signal are in bold and italics). As expected, overexpression of the miR-365-3p mutant had no significant effect on luciferase activity of this reporter compared to that in cells transfected with NC-miRNA. These findings indicate the existence of the interaction between miR-365-3p and the predicted binding site in the porcine *PLET1-L* 3′ UTR. However, unexpectedly, we also observed a significant decrease of the *PLET1-S* 3′ UTR luciferase reporter activity in cells transfected with miR-365-3p mimics compared to NC-miRNA. In addition, overexpression of the miR-365-3p mutant had no significant effect on the *PLET1-S* 3′ UTR luciferase reporter activity. The results suggest that a possible interaction may exist between *PLET1-S* 3′ UTR and miR-365-3p, although we did not find the perfect seed matches of miR-365-3p in the *PLET1-S* 3′ UTR by using the online miRNA target prediction tools described above. Due to the diverse nature of interactions between miRNA and mRNA, the importance of non-canonical matches (lacking perfect seed binding) are now widely appreciated [42,43]. However, the conventional computational methods we used for miRNA target prediction were seed-based, and focus on canonical matches (perfect seed binding). Therefore, our observation may suggest a non-canonical binding mechanism involved in the interaction between miR-365-3p and the *PLET1-S* 3′ UTR. Together with data shown in Figure 7, our study demonstrated the interaction between the *PLET1* and miR-365-3p in vitro, and indicates that in addition to the predicted canonical binding site in the *PLET1-L* 3′ UTR, a non-canonical miR-365-3p binding site might be within the *PLET1-S* 3′ UTR. Furthermore, our study found that the expression of miR-365-3p was significantly upregulated from Day 15 of gestation to Days 26, 50, and 95 of gestation, which shows similar expression pattern with the *PLET1-L* transcript as shown in Figure 5. This is opposed to the observations that miR-365-3p could reduce the *PLET1-L* 3′ UTR luciferase reporter activity in vitro and that the *PLET1-L* 3′ UTR showed higher luciferase activity. In this study, the expression data of miR-365-3p and *PLET1-L* 3′ UTR were derived from porcine placenta samples. Porcine placenta consists of not only trophoblast but also connective tissue. Our result indicates that *PLET1* is expressed exclusively in the trophoblast cells of porcine placenta, so Figure 5A shows the expression pattern of the *PLET1-L* transcript in the trophoblast cells. However, the cell location of miR-365-3p has yet to be assessed, so data shown in Figure 8 may not exactly be the expression pattern of miR-365-3p in trophoblast cells. This may explain the unexpected correlation of expression pattern between miR-365-3p and the *PLET1-L* transcript in porcine placenta. Therefore, further studies using porcine trophoblast cells will be needed to elucidate the detailed molecular mechanisms underlying the miR-365-3p-mediated regulation on the porcine *PLET1*.

## 4. Materials and Methods

### 4.1. Animals and Tissue Collections

All experimental procedures involving animals were approved by the Biological Studies Animal Care and Use Committee of Hubei Province and the Ethics Committee of Huazhong Agricultural University, China. Yorkshire gilts were obtained from the pig farm of Huazhong Agricultural University and were naturally mated at standing estrus to the same boar. Gilts were euthanized and slaughtered on Days 15, 26, 50, and 95 of gestation (*n* = 3 gilts/day of gestation). On Day 15 of gestation, pregnancy was confirmed by the presence of apparently normal filamentous conceptuses in uterine flushing, and the cross sections of uteri were then collected. On Days 26, 50 and 95 of gestation, the uteri were opened longitudinally along the antimesometrial side, and the cross sections of the utero-placental interface (including myometrium, endometrium, and placenta) in the implantation sites were collected as previously described [44]. These samples were fixed in 4% paraformaldehyde for 24 h followed by paraffin embedding. The conceptuses on Day 15 of gestation and placenta samples (chorioallantoic tissue) on Days 26, 50 and 95 of gestation were collected and stored in liquid nitrogen for RNA extraction.

### 4.2. Detection of the PLET1 mRNA Expression in Porcine Conceptus and Placenta by In Situ Hybridization

QuantiGene ViewRNA in situ hybridization was performed using procedures previously described using the QuantiGene ViewRNA FFPE Assay (Affymetrix/Panomics) [25]. Briefly, micrometer sections (4 μm thick) were cut and fixed in 10% formaldehyde for 1.5 h and then deparaffinized. Sections were boiled in a pre-treatment solution for 7 min and digested with protease QF for 15 min to give a pre-treatment before hybrid. Then, sections were hybridized for 3 h at 40 °C with a custom-designed QuantiGene ViewRNA probe against pig *PLET1*, the negative control was performed by replacing the probe with Hybridization Buffer A (Affymetrix). PreAmp and Amp molecules were used to amplify the bound probes, and multiple-labeled probe oligonucleotides conjugated to alkaline phosphatase were then added. The positive signals were present by adding FastRed substrate. Slides were counterstained with Gill’s Hematoxylin for 3 min at room temperature. Images were taken with a light microscope (Olympus BH-2, Tokyo, Japan).

### 4.3. Investigation of PLET1 Protein Expression in Porcine Conceptus and Placenta by Immunohistochemistry

To determine the expression of PLET1 in conceptus and placentas during the periods of pre-implantation (Day 15 of gestation) and placenta development (Days 26, 50, and 95 of gestation), IHC was performed by procedures as described [44]. Sections (4 μm thick) were deparaffinized with xylene and rehydrated in an alcohol gradient, and then incubated with 3% hydrogen peroxide (H_2_O_2_) to quench endogenous peroxidase for 15 min at room temperature. After rinsing with distilled water, the sections were boiled in 0.01 M sodium citrate buffer (pH 6.0) and cooled to room temperature twice to retrieve antigen. After rinsing with PBS, the sections were blocked with 5% bovine serum albumin (BSA) for 30 min and subsequently incubated with a custom-made Rabbit anti-Porcine PLET1 polyclonal antibody (Proteintech Corporation. Wuhan, China) (1:300) at 4 °C overnight, followed by an biotinylated secondary antibody (SA1022, Boster Corporation, Wuhan, China) (1:1000). The sections were counterstained with hematoxylin and mounted. For each sample, a negative control was performed by replacing the primary antibody with normal rabbit serum. All sections were stained immunohistochemically under the same conditions.

### 4.4. Investigation of the PLET1 Protein Expression in Porcine Conceptus and Placenta by Immunoflurescence 

Paraffin-embedded methacarn sections (4 μm thick) were deparaffinized with xylene and rehydrated in an alcohol gradient, and then washed twice in PBS. Nonspecific antibody-binding sites were blocked with 5% bovine serum albumin (BSA) for 1 h, and the slides were then rinsed in PBS. After overnight incubation with the Rabbit anti-Porcine PLET1 polyclonal antibody (1:300) at 4 °C, slides were rinsed in PBS and incubated in the secondary fluorescent Goat anti-Rabbit IgG-Cy3 (BA1032, Boster Corporation, Wuhan, China) (1:100) for 1 h at room temperature and rinsed in PBS, and slides were then stained with DAPI (blue) to identify nuclei. Sections were washed in PBS, and then cured with Anti-fade reagent (AR1109, Boster Corporation, Wuhan, China). The negative control was produced by replacing the primary antibody with normal rabbit serum. The results were observed and analyzed with a Nikon Eclipse E400epi-fluorescence microscope.

### 4.5. Cloning of the 5′and 3′ End of PLET1 Gene

Total RNA samples were extracted from conceptuses (Day 15 of gestation) and placentas (chorioallantoic tissue) respectively by using the Mini BEST universal RNA extraction kit (Takara Bio, Dalian, China). RNA was quantified by a Nanodrop 2000 spectrophotometer. The rapid amplification of cDNA ends (RACE) for mapping both the 5′ and 3′ cDNA ends was performed by using the SMARTer™ RACE cDNA Amplification Kit (Clontech Laboratories, Inc., Mountain View, CA, USA). The universal primer mix (UPM) was supplied by SMARTer™ RACE cDNA Amplification Kit and the sequences are listed in Table S1. The gene-specific primers for the 5′ and 3′ RACE reactions were also listed in Table S1. PCR products were purified and ligated to PMD19-T vector for sequencing.

### 4.6. Quantitative RT-PCR Analyses

The quantitative RT-PCR (qRT-PCR) was carried out to detect the expression levels of the two *PLET1* transcripts and the miR-365-3p expressed in the conceptuses (Day 15 of gestation) and placentas (chorioallantoic tissue, Days 26, 50, and 95 of gestation) on the CFX384 real-time PCR detection System (Bio-Rad Laboratories, Inc., Hercules, CA, USA). Total RNA was obtained as mentioned above. PCR was performed by using SYBR Premix Ex TaqII (Takara Bio, Dalian, China). The PCR amplifications were performed in triplicate for each sample. The significance test was carried out using the mixed linear model in the SAS 8.1 program. The model we used included the fixed effects of day of gestation (Days 15, 26, 50, and 95) and the random effect of piglet conceptus/placenta within gilt. A *p*-value of <0.05 was considered as significant. 

To detect the expression profiles of the two *PLET1* transcripts, the first strand cDNA was synthesized by using a PrimeScript RT reagent kit with a gDNA eraser (Takara Bio., Dalian, China). The primers were listed in Table S1. In order to detect the efficiency of primers, the sequential 1:4 dilutions of pooled cDNA were run in triplicate. Data from the serial dilutions of the control sample were used to calculate the PCR amplification efficiencies (E). The percent efficiency (Table S1) was calculated by the software provided by the CFX384 real-time PCR detection System (Bio-Rad Laboratories, Inc., Hercules, CA, USA). The thermal cycler was programed as follows: a single cycle of 30 s at 95 °C, followed by 45 cycles of 5 s at 95 °C, 30 s at 60 °C, and 15 s at 72 °C. The GAPDH gene was used as a control. 

To detect the expression profile of the miR-365-3p, total RNA was reverse transcribed using Mir-X^TM^ miRNA First-Strand Synthesis Kit (Takara Bio Inc., Dalian, China) according to the manufacturer’s instructions. DNA was removed by the Recombinant DNase I (RNase-free) (Takara Bio Inc., Dalian, China). Primers used were listed in Table S1. The RNU6 (small nuclear RNA U6) gene was used as a control. The thermal cycler was programed as follows: a single cycle for 5 min at 95 °C, followed by 45 cycles of 30 s at 95 °C, 20 s at 60 °C, and 15 s at 72 °C. The relative amount of miRNA to RNU6 was described using the equation 2^−ΔΔ*C*t^, where Δ*C*_t_ = (*C*_t miRNA_ − *C*_t RNU6_). The value of 2^−ΔΔ*C*t^ represents the relative expression level of miR-365-3p.

### 4.7. Construction of Porcine PLET1 3′UTRs Luciferase Reporter Vector and Luciferase Reporter Assay

Three constructs (designed as PLET1-S 3′ UTR, PLET1-L 3′ UTR, and PLET1-mutL 3′ UTR, respectively) were constructed to investigate the distinct *PLET1* 3′ UTRs activities. The total RNA isolated from the porcine placentas was used to synthesize the cDNA, and the cDNA was used to amplify the *PLET1-S* and *PLET1-L* 3′ UTRs, respectively. The first polyadenylation site-directed mutant was generated by overlap extension PCR method [45]. Primers used were listed in Table S1. Each of the segments was cloned into the psiCHECK-2 dual luciferase reporter plasmid (Promega, Madison, WI, USA) using the restriction enzymes *XhoI* (Fermentas, Waltham, MA, USA) and *NotI* (Fermentas). The sequences of the plasmid inserts were confirmed by DNA sequencing.

In the dual luciferase assays, PK15 cells were seeded in 96-well plates 24 h prior to transfection. To detect the different 3′ UTR activity of *PLET1*, psiCHECK-2-*PLET1-S*, psiCHECK-2-*PLET1-L*, and psiCHECK-2-*PLET1-mutL* plasmid were transfected into PK15 cells using Lipofectamine2000 (Invitrogen, Carlsbad, CA, USA) respectively, and cells were collected 24 h after transfection. Firefly and renilla luciferase activities were measured in cell lysates using a dual-luciferase reporter assay system (Promega), firefly luciferase activity was used as an internal control for the normalization of transfection efficiency, renilla/firefly luciferase activities were used to reflect the activity of *PLET1-S*, *PLET1-L*, and *PLET1-mutL* respectively. The transfections were repeated three times, and three replicates were performed for each transfection. A *p*-value of <0.05 by Student *t*-test was considered to be significant.

### 4.8. Validation of the Interaction between miR-365-3p and PLET1 

The two plasmids (PLET1-S and PLET1-L) that mentioned above and PLET1-L 3′ UTR plasmid where the putative miR-365-3p binding site was mutated (designed as PLET1-L-mut) were used to validate the interaction of miR-365-3p and *PLET1-L* 3′ UTR. The truncated wild-type segment (26 bp) that contained the predicted binding site was inserted into psiCHECK-2 by *Xho*I and *Not*I. The mutated binding site was introduced by the site-directed mutagenesis primers (listed in Table S1), and then cloned to psiCHECK-2 vector. The miRNA mimics of miR-365-3p, the miR-365-3p mutant (mut-miR-365-3p), and NC-miRNA (a scrambled sequence) were each synthesized as duplexes. Random 4 base substitutions in the seed sequence of miR-365-3p were introduced to create the miR-365-3p mutant (mut-miR-365-3p) when synthesized, and NC-miRNA was used as the negative control. All the sequences are listed in Table S1. The miRNA or mutant miRNA mimics (20 nM) or NC were co-transfected into PK15 cells with the reporter plasmid containing the corresponding binding sequences or mutant sequences using Lipofectamine 2000 as previously described [46]. The cell collection and the measurement of the dual-luciferase reporter activity have been described above. All the transfections were repeated three times, and three replicates were performed for each transfection. A *p*-value of <0.05 by Student’s *t*-test was considered as significant.

### 4.9. Protein Enzymatic Deglycosylation by PNGase F and endoH

Placentas (chorioallantoic tissue) from Yorkshire pig on Day 50 of gestation were used for protein extraction. Tissue was lysed with the RIPA lysis buffer (Beyotime, Shanghai, China), containing 1 mM phenyl methyl sulfonyl fluoride according to manufacturer’s instructions. Protein concentrations were measured using the BCA protein assay kit (Beyotime, Shanghai, China, P0012S). Protein (25 μg) was denatured with the denaturing buffer for 100 °C 10 min, and then samples were incubated at 37 °C for 16 h with two different glycosidases, peptide-*N*-glycosidase F (PNGase F, P0704S, NEB), and endoglycosidase H (EndoH, P0702S, NEB) according to the manufacturer’s protocol. A decrease in the molecular mass of PLET1 protein due to the removal of oligosaccharides was measured as change in migration distance after separation of the deglycosylated proteins on SDS-polyacrylamide gels, followed by western blot analysis as previously described [47]. The primary antibody specific for porcine PLET1 (1:300) as described above was used for incubation at 4 °C overnight. The HRP-labeled Goat anti-Rabbit IgG (1:1000) (Beyotime, Shanghai, China) was used as the secondary antibody.

## 5. Conclusions

In conclusion, our study revealed that PLET1 expression is restricted to the trophoblast cells in porcine placenta and its spatial localization in trophoblast cells differs before and after the establishment of the epitheliochorial placenta. In addition, two *PLET1* transcripts that differ in the 3′ UTR length were identified to be generated by the alternative cleavage and polyadenylation (APA). The expression of the *PLET1-L* transcript changed significantly before and after the establishment of the epitheliochorial placenta. Moreover, the present data suggest that miR-365-3p has an impact on the expression of the *PLET1* gene. Further investigation of the role of the alternative *PLET1* 3′ UTR isoforms on placenta development in pigs is needed.

## Supplementary Materials

Supplementary materials can be found at www.mdpi.com/1422-0067/17/12/2048/s1.
